# Decreased Rate of Evolution in Y Chromosome STR Loci of Increased Size of the Repeat Unit

**DOI:** 10.1371/journal.pone.0007276

**Published:** 2009-09-30

**Authors:** Mari Järve, Lev A. Zhivotovsky, Siiri Rootsi, Hela Help, Evgeny I. Rogaev, Elza K. Khusnutdinova, Toomas Kivisild, Juan J. Sanchez

**Affiliations:** 1 Department of Evolutionary Biology, University of Tartu and Estonian Biocentre, Tartu, Estonia; 2 Vavilov Institute of General Genetics, Russian Academy of Sciences, Moscow, Russia; 3 Brudnick Neuropsychiatric Research Institute, Department of Psychiatry, University of Massachusetts Medical School, Worcester, Massachusetts, United States of America; 4 Institute of Biochemistry and Genetics, Ufa Research Center, Russian Academy of Sciences, Ufa, Russia; 5 Leverhulme Centre for Human Evolutionary Studies, University of Cambridge, Cambridge, United Kingdom; 6 National Institute of Toxicology and Forensic Science, Canary Islands delegation, Campus de Ciencias de la Salud, La Laguna, Tenerife, Spain; Institute of Evolutionary Biology (CSIC-UPF), Spain

## Abstract

**Background:**

Polymorphic Y chromosome short tandem repeats (STRs) have been widely used in population genetic and evolutionary studies. Compared to di-, tri-, and tetranucleotide repeats, STRs with longer repeat units occur more rarely and are far less commonly used.

**Principal Findings:**

In order to study the evolutionary dynamics of STRs according to repeat unit size, we analysed variation at 24 Y chromosome repeat loci: 1 tri-, 14 tetra-, 7 penta-, and 2 hexanucleotide loci. According to our results, penta- and hexanucleotide repeats have approximately two times lower repeat variance and diversity than tri- and tetranucleotide repeats, indicating that their mutation rate is about half of that of tri- and tetranucleotide repeats. Thus, STR markers with longer repeat units are more robust in distinguishing Y chromosome haplogroups and, in some cases, phylogenetic splits within established haplogroups.

**Conclusions:**

Our findings suggest that Y chromosome STRs of increased repeat unit size have a lower rate of evolution, which has significant relevance in population genetic and evolutionary studies.

## Introduction

Y chromosome short tandem repeat (STR) markers are ever more commonly used in population genetic and evolutionary studies [1]–[3], genealogy research [4], [5] and human identification applications [6]. Y chromosome STRs, or microsatellites, consist of 1–6-bp units that are, on average, repeated 9.7 (nonpolymorphic loci) or 14.4 times (polymorphic loci) [7]. The number of new loci discovered in recent years is impressive [7], [8] and likely to grow even more. It has been claimed that applying machine-learning algorithms, Y chromosome STRs can be used to predict haplogroups of samples without the costly typing of SNP (single nucleotide polymorphism) markers [9]. Penta- and hexanucleotide repeats occur less frequently in the human genome and are so far less commonly employed in population genetic studies than di-, tri-, or tetranucleotide repeats.

While a recent study measured the Y chromosome base-substitution mutation rate as 3.0×10^−8^ mutations/nucleotide/generation [10], in the case of STRs, studies of deep rooting pedigrees have yielded an average Y-STR mutation rate of 2.0×10^−3^ per generation [11], which compares to the average rates of 2.5×10^−3^ [12] and 2.1×10^−3^ [13] per generation observed in father/son pairs. These so-called ‘pedigree’ rates have turned out to be an order of magnitude higher than the ‘evolutionary’ rate estimate of 2.6×10^−4^ per generation for the same STR loci, obtained in a study based on counting the number of mutations on the branches of a haplotype network [14]. This discrepancy might be explained by the fact that a large share of STR variation derived within a haplogroup is being effectively removed by genetic drift, rendering mutation rate estimates based on evolutionary considerations 3 or more times lower than those based on pedigree studies [15].

The effective mutation rate (based on evolutionary considerations) has been estimated as 1.52×10^−3^ per generation for an average autosomal dinucleotide STR locus and as 0.85−0.93×10^−3^ per generation for tri- and tetranucleotide loci [16]; the mutation rate for an average Y chromosome tri- or tetranucleotide STR locus has been estimated as 6.9×10^−4^ per 25 years [17]. These estimates set the mutation rate of dinucleotide STR loci about twice as high as that of tri- and tetranucleotide repeats. According to our knowledge, no estimate has been provided yet for the mutation rate of Y chromosome penta- or hexanucleotide STRs, although it is intuitively obvious that the figure should be lower than that of STR loci with smaller repeat unit sizes, since replication slippage, the mechanism of repeat count changes of STRs, is less likely to occur in case of longer repeats.

To estimate the scale of genetic variation of penta- and hexanucleotide STRs across diverse human populations and to compare the rate of evolution between STR loci with different repeat unit sizes, we have analysed 1 tri-, 14 tetra-, 7 penta-, and 2 hexanucleotide repeat loci within the male-specific region of the Y chromosome in 148 samples collected from diverse geographic regions and representing all the major Y chromosome haplogroups of the world (Table S1).

## Methods

### Ethics Statement

DNA samples from previously published sources were used, with the exception of Turkmens, Tajiks, and Bashkirs, which were collected with the approval of the Independent Ethics Committee of the Institute of Biochemistry and Genetics, Ufa Research Center, Russian Academy of Sciences (decision No 17/10.10.2007). Samples were obtained from unrelated volunteers after receiving written informed consent.

### Samples and DNA purification

A total of 148 unrelated male samples were typed (numbers in parentheses): Estonians (26), French (4), Slovaks (12), Romanians (1), Ukrainians (14), Caucasians (16), Turks (1), Iranians (8), Lebanese (2), Syrians (1), Egyptians (1), Ethiopians (1), Turkmens (3), Tajiks (3), Tatars (5), Russians (2), Maris (1), Bashkirs (7), Kazakhs (3), Khakashes (2), Altaians (14), Tuvas (5), Yakuts (1), Gujarat Indians (5), Punjab Indians (6), West Bengal Indians (1), Sri Lanka Moors (2), and Ijkas (1). Samples from populations analysed in [18] (Estonians, French), [19] (Slovaks, Romanians, Ukrainians, Turks, Iranians, Lebanese, Tatars, Russians), [20] (Caucasians, Maris, Kazakhs, Khakashes, Yakuts), [21] (Syrians, Egyptians, Ethiopians, Altaians, Tuvas), [22] (Indians, Sri Lanka Moors), and [23] (Ijkas) were used; other samples were obtained from Evgeny I. Rogaev (Turkmens, Tajiks) and Elza K. Khusnutdinova (Bashkirs). In addition, three female samples were included in the study to test for the specificity of the primers (controls).

DNA was purified from blood by phenol/chloroform, guanidinehydrochloride/proteinase K or methanol/NaOH/EDTANa_2_ extraction method. DNA concentrations were determined by spectrometry (NanoDrop products, Delaware, USA).

The samples represent all the major Y chromosome haplogroups of the world, having been typed for the defining SNP mutations in previous studies. The haplogroups (following the YCC nomenclature [24]) and defining mutations are reported in Table S1.

### Markers analysed, PCR conditions, capillary electrophoresis and sequencing

Seventeen of the markers analysed (1 tri-, 14 tetra-, 1 penta-, and 1 hexanucleotide STRs) belong to the AmpFlSTR® Yfiler™ Kit; the additional six penta- and one hexanucleotide STRs are reported in Table 1, five of them being previously described [7] and two novel.

**Table 1:** The markers analysed in this study not included in the AmpFlSTR® Yfiler™ Kit.

Marker	Repeat unit	GenBank accession number	Start	End	F primer sequences (5′>3′)	R primer sequences (5′>3′)
Y PENTA 1*	(AAAAC)_n_	AC010877	75633	75862	GGATTGAACTGTTTTGTCTTGGTG	gttTCAATCTTCAACCCACAGACC
DYF411S1**	(AAAGG)_n_(AAAG)_2_	AC068541	11073	11335	GTAATGACTGTGTTTGCACTTTCAC	gtttAAGCTTTTGTAAGTGTCATCCTAGC
DYS594	(AAATA)_n_	AC010137	50060	50279	AATTTAGATGTGCCTAATGCCACAG	gttTGAGTAACTTTCTGGCTCTTTTCC
DYS596	(GGA)_5_(GTA)_1_(GGA)_3_ (GAA)_3_(GGAGAA)_n_	AC016991	77103	77415	ATAACCGTGCCCTTTACTGC	GCCCAAAGTTCTTAACTTCCTTTTC
Y PENTA 2*	(TTCCA)_n_(TTCCG)_1_	AC069323	33200	33389	AGCTGATATTTCACTTCACCTTTCC	GGAATTGAAGGGAATGGATTTG
DYS643	(CTTTT)_n_	AC007007	25471	25908	AAGAAGTCACCATCCGTGAA	CTTTGGGAACTCAAGGGAAG
DYS645	(TGTTT)_n_(GAG)_2_	AC009239	14853	15235	GCAGCTTTTCCTTCTGTCAA	CTCTGCTTACCAATATCACTGC

Repeat units of the markers, GenBank accession numbers with the positions of the beginning of the forward primer and the end of the reverse primer in the GenBank sequence, and the primers used to amplify the markers. The ‘gtt’ or ‘gttt’ at the 5′ end of three of the reverse primers denotes a non-specific primer ‘tail’.

* novel markers.

** DYF411S1 was sequenced from the opposite strand of DNA compared to what was described by [7]. Complex repeats are presented as in [7], but only the variable penta/hexanucleotide repeats were counted (n repeats).

The samples were analysed with the Applied Biosystems AmpFlSTR® Yfiler™ Kit according to the recommendations of the manufacturer on the ABI PRISM® 3130*xl* Genetic Analyzer (Applied Biosystems, California, USA). The results were analysed using the ABI PRISM® program GeneMapper® 4.0 (Applied Biosystems).

The rest of the markers analysed in this study were found screening the human Y chromosome sequence in the GenBank database for penta- and hexanucleotide repeats, using Alex Dong Li's program RepeatFinder 0.4 (unfortunately no longer available, but there are similar programs, such as Tandem Repeats Finder, http://tandem.bu.edu/trf/trf.html) and looking for non-interrupted stretches of eight or more repeats. 41 Y-specific STRs were identified, 19 of them failed to amplify. Of the 22 remaining markers, 5 (Y PENTA 1, DYF411S1, DYS594, DYS596, Y PENTA 2) were analysed in a multiplex system, and 2 more (DYS643, DYS645) were genotyped for this study. The markers DYF411S1, DYS594, DYS596, DYS643, and DYS645 were previously described [7], whereas Y PENTA 1 and 2 were novel. The repeat units of the 7 penta- and hexanucleotide markers, the primers used to amplify them, and the GenBank accession numbers for the amplified regions are reported in Table 1. The forward primers of the five markers analysed in the multiplex system were labelled with fluorescent dyes at the 5′ ends: Y PENTA 1 and DYF411S1 with 6-FAM, DYS594 and DYS596 with HEX, and Y PENTA 2 with TAMRA.

The five STR markers amplified with fluorescence-labelled forward primers (Y PENTA 1, DYF411S1, DYS594, DYS596, Y PENTA 2) were amplified in a multiplex system under the following conditions: 1.25 µl GeneAmp PCR Buffer II without MgCl_2_, 1.5 µl MgCl_2_ (25 mM), 0.25 µl dNTP mix (10 mM), 2 µl PCR primer mastermix (individual primer concentrations 0.07–1.5 µM), 0.1 µl AmpliTaq Gold (5 U/µl), 6.4 µl ddH_2_O and 1 µl template DNA (1–10 ng/µl) were mixed per sample (total reaction volume 12.5 µl), and PCR cycling was performed as follows: 95°C, 10 min; 30 cycles (94°C, 30 sec; 60°C, 1 min; 72°C, 1 min); 65°C, 45 min; end at 10°C. Then, 0.5 µl of each PCR product and 0.15 µl of internal size standard (MegaBACE ET400-R Size Standard) were diluted in 9.5 µl Hi-Di Formamide and loaded directly onto the MicroAmp™ Optical 96-Well Reaction Plate. The samples were run on the ABI PRISM® 3130*xl* Genetic Analyzer (Applied Biosystems) using the Applied Biosystems Multi-Capillary DS-30 (Dye Set D) Matrix Std Kit as recommended by the manufacturer. The genotyping results were analysed using the ABI PRISM® programs GeneScan® 3.7 and Genotyper® 3.7 (both from Applied Biosystems).

Two STR markers (DYS643, DYS645) were amplified without fluorescent labels in separate PCR reactions under the following conditions: 1.5 µl GeneAmp PCR Buffer II without MgCl_2_, 1.2 µl MgCl_2_ (25 mM), 0.15 µl dNTP mix (10 mM), 2×0.3 µl PCR primer solution (10 µM each), 0.15 µl FIREPol® DNA Polymerase I (5 U/µl), 10.4 µl ddH_2_O and 1 µl template DNA (1–10 ng/µl) were mixed per sample (total reaction volume 15 µl) and PCR cycling was performed as follows: 94°C, 3 min; 40 cycles (94°C, 25 sec; 55°C, 30 sec; 72°C, 35 sec); 72°C, 3 min; end at 4°C. The products were sequenced using the Applied Biosystems BigDye® Terminator v3.1 Cycle Sequencing Kit as recommended by the manufacturer on the ABI PRISM® 3730*xl* DNA Analyzer (Applied Biosystems). The sequencing results were analysed using the program ChromasPro.

### Statistical analyses

Phylogenetic networks were constructed with the program Network 4.5.0.0, using the median joining algorithm.

The ability of the STR markers to differentiate haplogroups was tested with pairwise comparisons of repeat score distributions (p-values based on 10 000 permutations for exact Fisher test) between the haplogroups of the overrepresented R1 clade; the results of the penta/hexa and the tri/tetra markers were combined separately.

Repeat variance and sequence diversity [25] were calculated for all the markers, excluding the multicopy markers DYF411S1 and DYS385a/b, in which cases it was impossible to unambiguously distinguish the two copies. Both the repeat variance and diversity were averaged separately across the penta- and hexanucleotide markers and across the tri- and tetranucleotide markers in various data sets (Table 2). Average variance and diversity ratios between penta- and hexanucleotide STRs and tri- and tetranucleotide STRs were calculated (Table 2). The difference in the distribution of repeat variances within haplogroups between penta/hexa and tri/tetra markers was tested with the Mann-Whitney *U* test, using data from the R1 clade due to its larger sample size.

**Table 2:** Comparison of the average repeat variance and diversity between penta/hexa and tri/tetra markers.

	Source of data	Penta/hexa markers	Tri/tetra markers	Ratio between penta/hexa and tri/tetra
**Average repeat variance**	All data	0.513±0.091	0.922±0.167	0.557
	All data (R1a and R1b1b reduced)*	0.828±0.143	1.094±0.202	0.757
	R1a	0.223±0.058	0.373±0.072	0.597
	R1b1b2	0.132±0.040	0.440±0.174	0.300
	R1b1b1	0.204±0.126	0.635±0.279	0.320
	**Average**			**0.506**
**Average diversity**	All data	0.415±0.047	0.613±0.034	0.677
	All data (R1a and R1b1b reduced)*	0.600±0.040	0.678±0.028	0.886
	R1a	0.231±0.038	0.445±0.059	0.520
	R1b1b2	0.215±0.065	0.433±0.062	0.497
	R1b1b1	0.200±0.098	0.383±0.085	0.524
	**Average**			**0.621**

Multicopy markers DYF411S1 and DYS385a/b, in which cases it was impossible to unambiguously distinguish the two copies, were excluded from the calculations.

**Figure 1:**
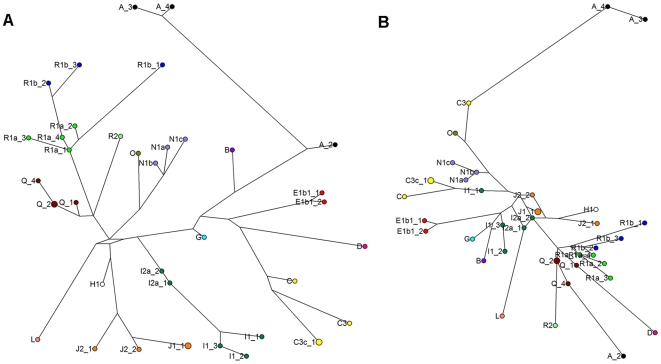
Networks of STR haplotypes based on penta- and hexanucleotide STRs, with and without SNPs. Median joining networks of Y chromosome STR haplotypes with balanced sample sizes from each haplogroup. A network based on 9 penta- and hexanucleotide STR markers and SNPs; B network based solely on the data of the 9 penta- and hexanucleotide STR markers used in this study. Nodes are named according to the haplogroups of the samples. STR markers employed in network construction: DYS448, DYS596, Y PENTA 1, Y PENTA 2, DYS438, DYS594, DYS643, DYS645, DYF411S1.

**Figure 2:**
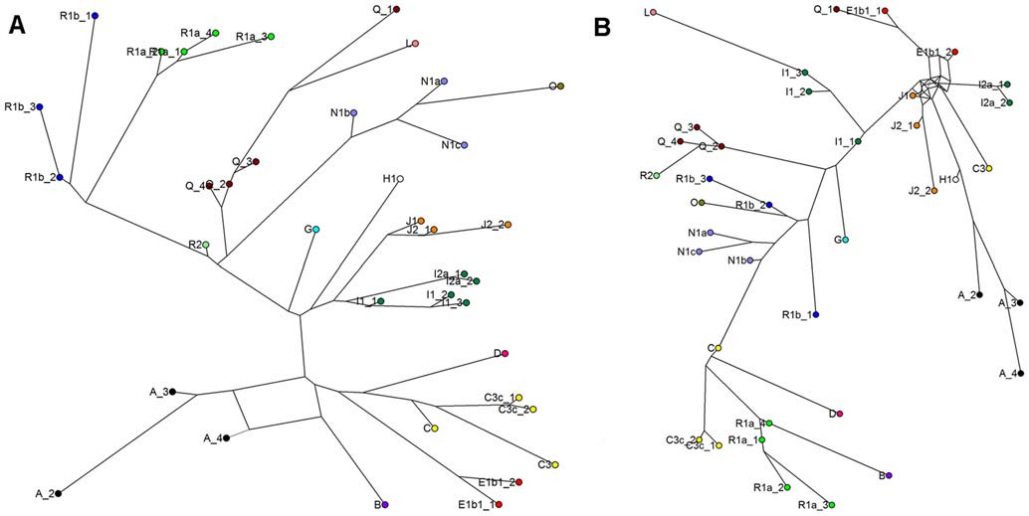
Networks of STR haplotypes based on tri- and tetranucleotide STRs, with and without SNPs. Median joining networks of Y chromosome STR haplotypes with balanced sample sizes from each haplogroup. A network based on 15 tri- and tetranucleotide STR markers and SNPs; B network based solely on the data of the 15 tri- and tetranucleotide STR markers used in this study. Nodes are named according to the haplogroups of the samples. STR markers employed in network construction: DYS19, DYS385a, DYS385b, DYS389I, DYS389II, DYS390, DYS391, DYS392, DYS393, DYS437, DYS439, DYS456, DYS458, DYS635, Y GATA H4.

* Haplogroups R1a and R1b1b were represented by the same samples as in Figures 1 and 2 (4 samples from R1a and 3 from R1b1b, marked with grey shading in Table S1).

Coalescence ages and their standard errors were calculated according to the ASD_0_ method [17], using penta- and hexanucleotide markers or tri- and tetranucleotide markers (Table 3). For the tri- and tetranucleotide markers, the previously estimated mutation rate of 6.9×10^−4^ per 25 years [17] was used, for the penta- and hexanucleotide markers, a two times lower rate of 3.45×10^−4^ per 25 years was used, based on the results of the present study.

**Table 3:** Coalescence age estimates and ancestral haplotypes of Y chromosome haplogroups.

	Haplogroup	Penta/hexanucleotide repeats: Y PENTA 1-DYS594-DYS596-Y PENTA 2-DYS643-DYS645-DYS438-DYS448	Tri/tetranucleotide repeats: DYS19-DYS389I-DYS389II-DYS390-DYS391-DYS392-DYS393-DYS437-DYS439-DYS456-DYS458-DYS635-Y GATA H4	SNP-based coalescence age estimates [24]
**Coalescence age estimate**	R1a	17,500±2,700	15,800±3,100	-
	R1b1b1	16,700(4,700	22,900(9,300	-
	R1b1b2	10,900(1,800	16,600(6,000	-
	R1	30,900(3,300	31,900(6,200	-
	R1 (Europe, 14 R1a+14 R1b1b2)	23,300(4,300	27,000(5,500	18,500 (12,500–25,700)
	R (8 balanced samples)	39,600(5,300	41,800(11,400	26,800 (19,900–34,300)
	P (8 R+4 Q)	31,700(4,500	41,300(8,100	34,000 (26,600–41,400)
	K (12 P+4 NO+1 L)	42,100(3,900	42,600(9,200	47,400 (40,000–53,900)
	F (27 samples, incl 17 K)	43,600(3,100	46,000(10,000	48,000 (38,700–55,700)
	CF	64,700(5,700	42,200(7,200	68,900 (64,600–69,900)
**Ancestral haplotype**	R1a	11-10-10-10-10-8-11-20	16-13-17-25-11-11-13-14-10-16-15-23-12	
	R1b1b1	13-10-10-10-9-8-10-19	14-14-17-21-11-13-13-15-13-15-16-23-11	
	R1b1b2	11-10-10-11-10-8-12-19	14-13-16-24-11-13-13-15-12-16-17-23-12	
	R1	11-10-10-10-10-8-11-19	15-13-17-24-11-12-13-15-11-16-16-23-12	
	R1 (Europe, 14 R1a+14 R1b1b2)	11-10-10-10-10-8-11-20	15-13-16-24-11-12-13-15-11-16-16-23-12	
	R (8 balanced samples)	11-10-10-10-10-8-11-19	15-13-16-24-11-12-13-15-12-15-17-23-12	
	P (8 R+4 Q)	11-10-10-10-10-8-11-19	15-14-16-24-10-11-13-15-11-15-17-23-12	
	K (12 P+4 NO+1 L)	11-10-9-10-10-8-10-19	15-13-16-23-10-13-13-15-11-15-17-22-12	
	F (27 samples, incl 17 K)	11-10-9-10-10-8-10-20	15-13-16-23-10-11-13-15-12-15-16-21-12	
	CF	11-11-10-9-10-8-10-20	15-13-16.5-24-10-11-13-14-12-15-17-22-11	

Coalescence age estimates, based on penta/hexanucleotide and tri/tetranucleotide repeats and the respective mutation rates, and ancestral haplotypes (estimated as the weighted median number of repeats at each locus) of Y chromosome haplogroups. SNP-based age estimates from [24] are reported for comparison. Multicopy markers DYF411S1 and DYS385a/b were excluded from the calculations.

Time series of STR locus variances were compiled in the growing order of haplogroup variances relative to the age estimates provided by [24]. Time-dependent behaviour of each marker (excluding the multicopy markers DYF411S1 and DYS385a/b) was characterised by the value of α, the proportion of the average variance of the younger versus the older clades relative to their respective age estimates (Table 4, α = [mean variance(R1a, R1b1b2)/mean variance(P,K,F)]/[age(R1a, R1b1b2)/age(P,K,F)]). Spearman rank correlations were also calculated, using the SPSS 14.0 package (Table 4).

**Table 4:** Temporal dynamics of different STR loci–time series of STR locus variances by haplogroup age estimates.

	SNP age (ky) [24]	Relative age	DYS392	*Y PENTA 1*	DYS437	DYS390	*DYS645*	*DYS596*	DYS19	DYS635	*Y PENTA 2*	DYS389II	DYS393	*DYS643*	*DYS438*	DYS439	*DYS594*	DYS389I	YGATA H4	*DYS448*	DYS456	DYS458	DYS391	Average	Standard deviation
**R1a**	-	**0.29**	0.05	*0.06*	0.05	0.37	*0.02*	*0.16*	0.36	0.10	*0.20*	0.50	0.11	*0.23*	*0.10*	0.44	*0.54*	0.34	0.51	*0.46*	0.86	0.80	0.38	**0.32**	**0.24**
**R1b1b1**	-	**0.35**	0.00	*0.44*	0.00	3.47	*0.00*	*0.00*	0.00	0.27	*0.00*	1.81	0.08	*1.00*	*0.00*	0.47	*0.00*	0.56	0.00	*0.19*	0.19	1.14	0.26	**0.47**	**0.83**
**R1b1b2**	-	**0.24**	0.11	*0.00*	0.05	0.05	*0.00*	*0.00*	0.09	0.47	*0.24*	0.45	0.20	*0.27*	*0.21*	0.41	*0.09*	0.34	0.26	*0.24*	0.54	2.45	0.30	**0.32**	**0.51**
**R1**	-	**0.52**	0.86	*0.76*	0.23	2.39	*0.02*	*0.12*	0.90	0.21	*0.26*	0.76	0.13	*0.44*	*0.39*	1.35	*0.43*	0.38	0.54	*0.48*	0.76	1.36	0.35	**0.62**	**0.55**
**R1** 1	**18.5**	**0.41**	1.26	*0.04*	0.26	0.40	*0.04*	*0.04*	1.07	0.30	*0.46*	0.70	0.30	*0.27*	*0.54*	1.18	*0.04*	0.25	0.18	*0.76*	0.77	1.96	0.34	**0.53**	**0.50**
**R** 2	**26.8**	**0.67**	1.36	*1.36*	0.55	4.79	*0.00*	*0.13*	1.36	0.21	*0.98*	1.84	0.13	*0.79*	*0.29*	1.13	*0.70*	0.55	0.50	*0.57*	0.29	1.36	0.27	**0.91**	**1.03**
**P** 3	**34**	**0.70**	1.94	*1.08*	0.74	2.92	*0.00*	*0.14*	1.33	0.59	*0.74*	1.26	0.14	*0.47*	*0.17*	0.92	*0.50*	0.44	0.47	*0.33*	0.44	0.90	0.27	**0.75**	**0.68**
**K** 4	**47.4**	**0.85**	3.22	*0.86*	0.69	2.57	*0.13*	*0.63*	1.15	1.43	*0.94*	1.13	0.50	*0.50*	*0.35*	0.88	*0.50*	0.49	0.36	*0.25*	0.51	1.26	0.22	**0.89**	**0.76**
**F** 5	**48**	**0.80**	2.36	*0.56*	0.69	1.79	*0.08*	*0.46*	0.91	1.78	*0.64*	1.10	0.49	*0.85*	*0.54*	0.92	*0.67*	0.49	0.48	*0.38*	0.71	1.37	0.22	**0.83**	**0.57**
**CF**	**68.9**	**1.00**	2.62	*0.87*	0.69	1.75	*0.15*	*0.60*	0.97	1.53	*0.67*	0.98	0.45	*1.06*	*0.46*	0.90	*0.76*	0.44	0.56	*0.62*	0.73	1.41	0.27	**0.88**	**0.56**
**Spearman rank correlation**			.93**	.*73* *	.86**	.37	.*65* *	.*80* **	.62	.68*	.*76* *	.47	.66*	.*66* *	.*52*	.35	.*63*	.42	.41	.*27*	−.14	.01	−.60		
**Coefficient of age prediction from variance α**			0.09	*0.12*	0.21	0.26	*0.53*	*0.56*	0.59	0.65	*0.85*	1.21	1.21	*1.24*	*1.29*	1.38	*1.68*	2.15	2.56	*3.21*	3.71	4.06	4.24		

Penta- and hexanucleotide markers shown in italics. Multicopy markers DYF411S1 and DYS385a/b were excluded from the calculations.

1 Europe, 14 R1a+14 R1b1b2 samples.

2 8 balanced samples.

3 8 R+4 Q samples.

4 12 P+4 NO+1 L samples.

5 27 samples, incl 17 K.

α–proportion of the average variance of the younger (<.3) versus older (≥.7) clades relative to their respective age estimates. α = [mean variance(R1a, R1b1b2)/mean variance(P,K,F)]/[age(R1a, R1b1b2)/age(P,K,F)].

* p<0.05.

** p<0.01.

## Results

We analysed 1 tri-, 14 tetra-, 7 penta-, and 2 hexanucleotide STR markers within the male-specific region of the human Y chromosome in 148 samples collected from diverse geographic regions and belonging to a broad range of Y chromosome haplogroups (Table S1) in order to evaluate genetic variation in STRs with different repeat unit sizes. Our study included too few tri- and hexanucleotide markers to make any definitive statements about them, but we grouped them together with tetra- and pentanucleotide markers, respectively, due to similar behaviour.

To compare the ability of STR loci with different repeat unit sizes to distinguish Y chromosome haplogroups, we constructed median joining phylogenetic networks based on a data set in which each haplogroup was represented by 1–4 individual samples (4 samples from haplogroup R1a and 3 from R1b1b, marked with grey shading in Table S1). Networks were constructed based on the 9 penta- and hexanucleotide STRs (Figure 1) and based on the 15 tri- and tetranucleotide STRs (Figure 2), providing both networks that included SNP markers in their construction (Figures 1a and 2a) and those that did not (Figures 1b and 2b).

The network based solely on the 9 penta- and hexanucleotide STR markers (Figure 1b) generally grouped haplotypes well together according to their SNP-based haplogroup affiliations. However, the internal hierarchy of the branches of the SNP- and STR-based trees showed only weak correlation (Figure 1). Similarly, the network based on the tri- and tetranucleotide STR markers (Figure 2b) showed a clustering of haplotypes according to their SNP-defined haplogroups (e.g. haplogroups A and R1a), but a low level of concordance in the internal relationships of the haplogroups (Figure 2). Despite using a higher number of markers (15), the tri- and tetranucleotide network was, unlike that based on 9 penta- and hexanucleotide STR markers, unable to establish, for example, the sister-clade status of haplogroups R1a and R1b1b, or to reconstruct haplogroup N as a monophyletic clade. Statistical analyses (Fisher test pairwise comparisons of repeat score distributions between haplogroups) indicate that both penta/hexa and tri/tetra STR markers are well capable of distinguishing haplogroups without SNP marker data; in practice, however, the network based on penta/hexa markers reflects the haplogroup affiliations of haplotypes better.

**Figure 3:**
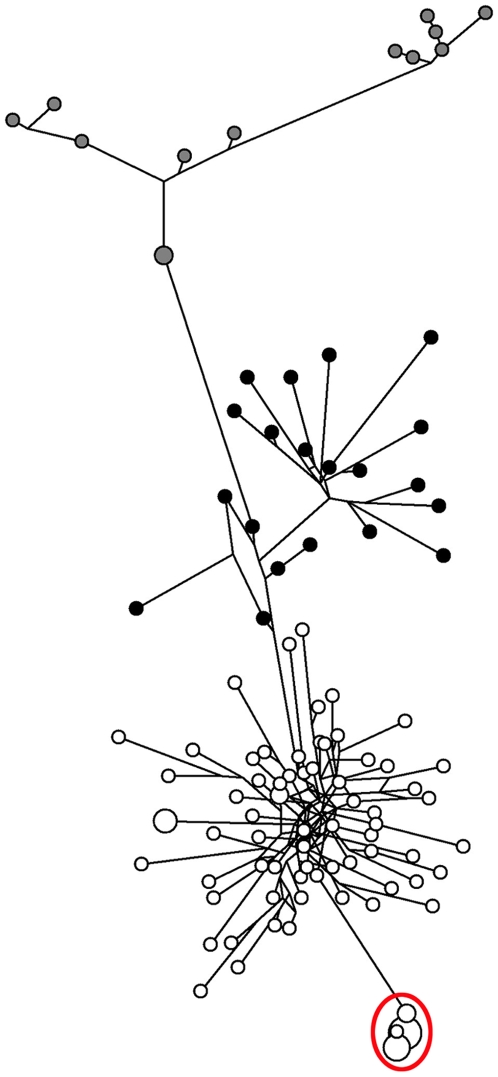
Network of R1a and R1b1b STR haplotypes based on the data of all the markers. Median joining network of all the samples belonging to haplogroups R1a and R1b1b, based on the data of all the 24 markers used in this study. Open circles represent haplotypes of haplogroup R1a, black those of haplogroup R1b1b2, grey those of haplogroup R1b1b1. 13 nearly identical Altaian and Tuva samples form a separate branch within R1a, indicated by a red circle.

Due to their large sample sizes, in the case of sister haplogroups R1a (n = 82) and R1b1b (n = 33), combined data of all the markers was used to obtain a high resolution median joining network (Figure 3). Most haplotypes in this network are represented by a single individual. However, it is notable that inside haplogroup R1a (represented by open circles in Figure 3), several individual samples still exhibit identical haplotypes even at the resolution of 24 Y-STR markers. A separate branch of nearly identical Altaian and Tuva samples from haplogroup R1a can be seen to emerge (marked by a red circle in Figure 3), indicating that STR marker data can be used to point to potential intra-haplogroup subdivisions. This is further demonstrated by the clear separation of sister clades R1b1b2 (n = 20, represented by black circles in Figure 3) and R1b1b1 (n = 13, represented by grey circles) within haplogroup R1b1b. However, this division, as well as the high intrahaplogroup variability of R1b1b1, is not surprising, since unlike R1b1b2, R1b1b1 is a low frequency ancient haplogroup, the haplotype structure of which has apparently been significantly influenced by genetic drift.

Repeat variance and diversity were calculated for all the markers except DYF411S1 and DYS385a/b, in which cases it was impossible to unambiguously distinguish the alleles at two different copies. Both the average variance and the average diversity of penta- and hexanucleotide markers were lower than those of tri- and tetranucleotide STRs (Table 2). The average repeat variance and diversity values with standard errors were calculated not only for the whole data, but also separately for the data set with balanced sample sizes from each haplogroup and for the overrepresented R1 clade (haplogroups R1a, R1b1b2 and R1b1b1), and the ratios calculated showed that penta/hexa variation is on average two times lower than tri/tetra variation (Table 2). Because interhaplogroup comparisons of locus variances might be biased due to different ancestral repeat lengths, the difference in the distribution of repeat variances within haplogroups between penta/hexa and tri/tetra markers was tested using the data of the three closely related R1 clade haplogroups (R1a, R1b1b1, and R1b1b2) with extended sample sizes. The p-value of the combined Fisher test on the three p-values from the Mann-Whitney *U* test of distribution was 0.0047, confirming the alternative hypothesis that the median of the penta/hexa variances is smaller than that of the tri/tetra variances. In order to obtain comparable coalescence time estimates for Y chromosome haplogroups, we therefore employed a mutation rate of 3.45×10^−4^ per 25 years for the penta/hexa markers (Table 3), which is two times lower than the estimate of 6.9×10^−4^ per 25 years for the tri/tetra loci [17].

The STR markers employed were assessed regarding their clock-like behaviour, characterised by the value of α, the proportion of the average variance of the younger versus the older clades relative to their respective age estimates (Table 4, α = [mean variance(R1a, R1b1b2)/mean variance(P,K,F)]/[age(R1a, R1b1b2)/age(P,K,F)]). The coefficient of age prediction from variance α thus describes the concordance of the mean variance of an STR marker with the age estimates of younger versus older clades. The variance of a clock-like marker would be expected to increase with haplogroup age and in case of a linear relationship α would be approximately 1. Comparing the temporal dynamics of the STR loci analysed (Table 4), 6 of the 8 penta- and hexanucleotide markers behaved more or less clock-like (α = 0.5–1.7, Table 4), whereas only 5 of the 13 tri- and tetranucleotide markers fell into the same category–on one extreme, DYS392, while showing high interhaplogroup variances, demonstrated virtually no variance in young haplogroups; on the other extreme, DYS391 showed equal or higher variances in young haplogroups relative to old ones, likely because of saturation of mutation events between its two modal repeat count states. Spearman's rank test was also performed to evaluate the correlation between clade age and marker variance, but there is an essential difference between Spearman's correlation coefficients and α, the latter taking into account not only the rank of the estimates in the array, but also their relative values. For example, in the case of DYS392, the Spearman correlation between clade age and variance is strongly positive and significant, whereas based on α, the ratio of variances between younger and older clades does not correlate strongly with the ratio of clade ages (i.e. the marker does not behave in a clock-like manner).

## Discussion

Most of the STR markers used in the population and evolutionary studies of the human Y chromosome have been tri- or tetranucleotide repeats (e.g. in the Applied Biosystems AmpFlSTR® Yfiler™ Kit and the PowerPlex® Y System). Given the relatively lower mutation rates of tri- and tetranucleotide STRs compared to dinucleotide loci, it is theoretically plausible that the penta- and hexanucleotide repeats evolve at a lower rate than tri- and tetranucleotide repeats, although still much faster than SNPs. They should therefore prove to be an attractive class of STR markers to be used in Y chromosome population and forensic relationship testing studies.

If a population is at mutation-drift equilibrium, the variance at an STR locus is proportional to the (effective) mutation rate [17]. In equilibrium, the variance ratio between penta/hexa and tri/tetra STRs times a mutation rate of tri- and tetranucleotide markers would give a mutation rate of penta- and hexanucleotide STRs. However, variation within any haplogroup in any human population is far from equilibrium. An estimate that would represent the effective mutation rate among the penta- and hexanucleotide markers studied is within-population within-haplogroup STR variation averaged across various populations and haplogroups. Bearing this in mind, it is important to use as much data as possible in order to obtain the entire ranges of Y-STR variation. For this reason, we included 115 samples from the R1 clade with two common haplogroups showing opposite clinal patterns [26], [27] in Europe–R1a and R1b1b2, and one rare haplogroup that has apparently gone through bottlenecks and/or founder effects–R1b1b1. It can be seen that both the average repeat variance and the average diversity vary considerably between different data sets and haplogroups within our data (Table 2); therefore, obviously, studies with larger data sets would improve on our results. Nevertheless, this study shows consistent average repeat variance and diversity ratios of approximately 0.5 between penta/hexa and tri/tetra markers, which allows us to estimate that the average mutation rate of penta- and hexanucleotide STRs is around a half of that of tri- and tetranucleotide STRs. The major contributors to this difference are penta- and tetranucleotide markers, we cannot draw any conclusions from hexa- and trinucleotide markers due to too small numbers of loci. Overall, we notice a trend that STRs of increased size of the repeat unit exhibit lower variation.

Since repeat complexity and repeat count (in case of complex STRs, the repeat count of the longest homogenous array) have also been reported to influence STR marker variation [7], we analysed our markers according to these features in order to ascertain whether the difference observed between tri/tetra and penta/hexa marker variation was indeed due to repeat unit size. Based on the limited number of markers included in the present study, repeat variance and diversity averaged across simple versus complex repeats (disregarding repeat unit size) showed hardly any difference at all, whereas repeat count did seem to have an effect on marker variation, especially on repeat variance (higher repeat variance corresponding to higher repeat count), the latter observation confirming previous results [7]. Our data set and that of [7] are not well comparable, the latter having a large number of loci and a small number of samples, whereas we have a small number of loci and a larger number of samples, and we cannot state definitively whether STR marker variation depends on repeat unit size or repeat count (or both). However, sequence composition has no effect on STR variation, since neither Student's nor Welch's *t* test showed any significant difference in the sequence composition of penta/hexa versus tri/tetra markers (calculating the proportions of the nucleotides in the repeats and considering that A = T and G = C, p>0.2 for each test).

In order to compare age estimates based on tri- and tetranucleotide versus penta- and hexanucleotide markers, coalescence ages of Y chromosome haplogroups were calculated based on both the tri/tetra and the penta/hexa STR results, using the previously estimated mutation rate of 6.9×10^−4^ per 25 years [17] for the tri/tetra markers and a two times lower mutation rate of 3.45×10^−4^ per 25 years for the penta/hexa markers. For our calculations, different sample sets representing various Y chromosome clades were assembled to compare the age estimates of tri/tetra or penta/hexa STRs to SNP-based estimates [24]. The results (Table 3) show that in most cases, coalescence age estimates based on the tri/tetra and penta/hexa marker clocks are comparable, although the error margins are rather wide. While within the R clade the SNP-based age estimate is, as expected, lower than the STR-based estimates, it is greater than the STR-based estimates for the older clades K, F, and CF (Table 3). This indicates STR locus saturation, which seems to occur more rapidly in case of tri- and tetranucleotide markers (the age estimate for the CF clade based on tri/tetra marker results is 42,200 years, considerably lower than the estimate of 64,700 years based on penta/hexa marker results and the estimate of 68,900 years based on SNP marker results [24]). On the whole, absolute age estimates vary considerably and are therefore rather unreliable, while relative age estimates show patterns more consistent with the relative age distribution of SNP-defined haplogroups.

The penta- and hexanucleotide markers analysed were relatively more clock-like in their behaviour (α = 0.5–1.7, Table 4) than the tri- or tetranucleotide loci in their variance time series. DYS392, Y PENTA 1, and DYS437 were not variable enough to be informative within a time frame of 20,000 years, particularly considering our limited sample sizes; on the other hand, DYS456, DYS458, and DYS391 appeared to be quickly saturated (Table 4). The generally clock-like behaviour of penta- and hexanucleotide markers underlines their applicability in evolutionary studies.

Based on our results, penta- and hexanucleotide STR markers surpass tri- and tetranucleotide markers in the ability to distinguish Y chromosome haplogroups without SNP data (Figures 1 and 2). Their ability to group samples according to their haplogroups is confirmed by the results of the combined Fisher test showing significant differences in repeat score distributions of penta/hexa loci between different haplogroups. Although the establishment of reliable phylogenetic relations requires additional SNP marker data, STRs can be used to distinguish Y chromosome haplogroups and, in some cases, subdivisions within haplogroups, as we show in this study for R1a and R1b1b (Figure 3). Our findings show that in some cases, samples can be accurately assigned to Y chromosome haplogroups based solely on Y-STRs, corroborating the conclusion of a recent study [9].

In conclusion, our results show that STRs of increased repeat unit size have a lower rate of evolution. This must naturally be taken into account when estimating STR mutation rates, and along with the slower locus saturation and the generally clock-like behaviour exhibited by the penta- and hexanucleotide markers analysed in this study, it makes STRs with longer repeat units well applicable in population and evolutionary studies, perhaps even more so than their counterparts with shorter repeat units.

## Supporting Information

Table S1 Samples and STR markers analysed. The samples representing haplogroups R1a and R1b1b in the data set with balanced sample sizes from each haplogroup (used in Figures 1 and 2) are marked with grey shading. In the case of DYF411S1, when only one repeat number is shown, only one product was observed, but this is believed to be due to two products of the same size overlapping, and thus two equal repeat numbers are assumed.
(0.07 MB XLS)

## References

[1] de Knijff P (2000). Messages through bottlenecks: on the combined use of slow and fast evolving polymorphic markers on the human Y chromosome.. Am J Hum Genet.

[2] Kayser M, Krawczak M, Excoffier L, Dieltjes P, Corach D (2001). An extensive analysis of Y-chromosomal microsatellite haplotypes in globally dispersed human populations.. Am J Hum Genet.

[3] Jobling MA, Tyler-Smith C (2003). The human Y chromosome: an evolutionary marker comes of age.. Nat Rev Genet.

[4] Zerjal T, Xue Y, Bertorelle G, Wells RS, Bao W (2003). The genetic legacy of the Mongols.. Am J Hum Genet.

[5] Xue Y, Zerjal T, Bao W, Zhu S, Lim SK (2005). Recent spread of a Y-chromosomal lineage in northern China and Mongolia.. Am J Hum Genet.

[6] Hanson EK, Berdos PN, Ballantyne J (2006). Testing and evaluation of 43 “noncore” Y chromosome markers for forensic casework applications.. J Forensic Sci.

[7] Kayser M, Kittler R, Erler A, Hedman M, Lee AC (2004). A comprehensive survey of human Y-chromosomal microsatellites.. Am J Hum Genet.

[8] Hanson EK, Ballantyne J (2006). Comprehensive annotated STR physical map of the human Y chromosome: Forensic implications.. Leg Med (Tokyo).

[9] Schlecht J, Kaplan ME, Barnard K, Karafet T, Hammer MF (2008). Machine-learning approaches for classifying haplogroup from Y chromosome STR data.. PLoS Comput Biol.

[10] Xue Y, Wang Q, Long Q, Ng BL, Swerdlow H (2009). Human Y Chromosome Base-Substitution Mutation Rate Measured by Direct Sequencing in a Deep-Rooting Pedigree.. Curr Biol.

[11] Heyer E, Puymirat J, Dieltjes P, Bakker E, de Knijff P (1997). Estimating Y chromosome specific microsatellite mutation frequencies using deep rooting pedigrees.. Hum Mol Genet.

[12] Goedbloed M, Vermeulen M, Fang RN, Lembring M, Wollstein A (2009). Comprehensive mutation analysis of 17 Y-chromosomal short tandem repeat polymorphisms included in the AmpFlSTR(R) Yfiler(R) PCR amplification kit.. Int J Legal Med.

[13] Ge J, Budowle B, Aranda XG, Planz JV, Eisenberg AJ (2009). Mutation rates at Y chromosome short tandem repeats in Texas populations.. Forensic Sci Int Genet.

[14] Forster P, Rohl A, Lunnemann P, Brinkmann C, Zerjal T (2000). A short tandem repeat-based phylogeny for the human Y chromosome.. Am J Hum Genet.

[15] Zhivotovsky LA, Underhill PA, Feldman MW (2006). Difference between evolutionarily effective and germ line mutation rate due to stochastically varying haplogroup size.. Mol Biol Evol.

[16] Zhivotovsky LA, Bennett L, Bowcock AM, Feldman MW (2000). Human population expansion and microsatellite variation.. Mol Biol Evol.

[17] Zhivotovsky LA, Underhill PA, Cinnioglu C, Kayser M, Morar B (2004). The effective mutation rate at Y chromosome short tandem repeats, with application to human population-divergence time.. Am J Hum Genet.

[18] Tambets K, Rootsi S, Kivisild T, Help H, Serk P (2004). The western and eastern roots of the Saami–the story of genetic “outliers” told by mitochondrial DNA and Y chromosomes.. Am J Hum Genet.

[19] Rootsi S, Magri C, Kivisild T, Benuzzi G, Help H (2004). Phylogeography of Y-chromosome haplogroup I reveals distinct domains of prehistoric gene flow in europe.. Am J Hum Genet.

[20] Rootsi S, Zhivotovsky LA, Baldovic M, Kayser M, Kutuev IA (2007). A counter-clockwise northern route of the Y-chromosome haplogroup N from Southeast Asia towards Europe.. Eur J Hum Genet.

[21] Reidla M, Kivisild T, Metspalu E, Kaldma K, Tambets K (2003). Origin and diffusion of mtDNA haplogroup X.. Am J Hum Genet.

[22] Kivisild T, Rootsi S, Metspalu M, Mastana S, Kaldma K (2003). The genetic heritage of the earliest settlers persists both in Indian tribal and caste populations.. Am J Hum Genet.

[23] Tamm E, Kivisild T, Reidla M, Metspalu M, Smith DG (2007). Beringian standstill and spread of Native American founders.. PLoS One.

[24] Karafet TM, Mendez FL, Meilerman MB, Underhill PA, Zegura SL (2008). New binary polymorphisms reshape and increase resolution of the human Y chromosomal haplogroup tree.. Genome Res.

[25] Nei M (1987). Molecular evolutionary genetics..

[26] Rosser ZH, Zerjal T, Hurles ME, Adojaan M, Alavantic D (2000). Y-chromosomal diversity in Europe is clinal and influenced primarily by geography, rather than by language.. Am J Hum Genet.

[27] Semino O, Passarino G, Oefner PJ, Lin AA, Arbuzova S (2000). The genetic legacy of Paleolithic Homo sapiens sapiens in extant Europeans: a Y chromosome perspective.. Science.

